# *In vivo* Delivery Tools for Clustered Regularly Interspaced Short Palindromic Repeat/Associated Protein 9-Mediated Inhibition of Hepatitis B Virus Infection: An Update

**DOI:** 10.3389/fmicb.2022.953218

**Published:** 2022-07-01

**Authors:** Mohammad Enamul Hoque Kayesh, Md Abul Hashem, Michinori Kohara, Kyoko Tsukiyama-Kohara

**Affiliations:** ^1^Joint Faculty of Veterinary Medicine, Transboundary Animal Diseases Centre, Kagoshima University, Kagoshima, Japan; ^2^Department of Microbiology and Public Health, Faculty of Animal Science and Veterinary Medicine, Patuakhali Science and Technology University, Barishal, Bangladesh; ^3^Department of Microbiology and Cell Biology, Tokyo Metropolitan Institute of Medical Science, Tokyo, Japan

**Keywords:** hepatitis B virus, cccDNA, CRISPR/Cas9, delivery, gene therapy

## Abstract

Chronic hepatitis B virus (HBV) infection remains a major global health problem despite the availability of an effective prophylactic HBV vaccine. Current antiviral therapies are unable to fully cure chronic hepatitis B (CHB) because of the persistent nature of covalently closed circular DNA (cccDNA), a replicative template for HBV, which necessitates the development of alternative therapeutic approaches. The CRISPR/Cas system, a newly emerging genome editing tool, holds great promise for genome editing and gene therapy. Several *in vitro* and/or *in vivo* studies have demonstrated the effectiveness of HBV-specific clustered regularly interspaced short palindromic repeat (CRISPR)/associated protein 9 (CRISPR/Cas9) systems in cleaving HBV DNA and cccDNA. Although recent advances in CRISPR/Cas technology enhance its prospects for clinical application against HBV infection, *in vivo* delivery of the CRISPR/Cas9 system at targets sites remains a major challenge that needs to be resolved before its clinical application in gene therapy for CHB. In the present review, we discuss CRISPR/Cas9 delivery tools for targeting HBV infection, with a focus on the development of adeno-associated virus vectors and lipid nanoparticle (LNP)-based CRISPR/Cas ribonucleoprotein (RNP) delivery to treat CHB. In addition, we discuss the importance of delivery tools in the enhancement of the antiviral efficacy of CRISPR/Cas9 against HBV infection.

## Introduction

Genome editing is a novel approach used for manipulating target genes in various cell types and organisms using engineered nucleases. Zinc finger nucleases (ZFNs), transcription activator-like effector nucleases (TALENs), and clustered regularly interspaced short palindromic repeat (CRISPR)/associated protein 9 (CRISPR/Cas9) nuclease systems are some of the genome-editing tools that have been extensively studied (Gaj et al., 2013). The CRISPR/Cas9 nuclease system has emerged as a potent genome editing tool and has revolutionized genome editing (Jinek et al., 2012; Cong et al., 2013; Doudna and Charpentier, 2014; Knott and Doudna, 2018; Pickar-Oliver and Gersbach, 2019). CRISPR/Cas9, a naturally occurring RNA-guided endonuclease, has been primarily discovered in bacteria and serves as a defense tool for adaptive immunity against bacteriophages (Bolotin et al., 2005; Mojica et al., 2005; Pourcel et al., 2005; Barrangou et al., 2007). Compared to ZFNs and TALENs, the CRISPR/Cas9 system is simple, efficient, and readily reprogrammable, and different DNA sequences can be targeted simply by redesigning guide RNAs (gRNAs) (Cong et al., 2013; Hu et al., 2016).

**FIGURE 1:**
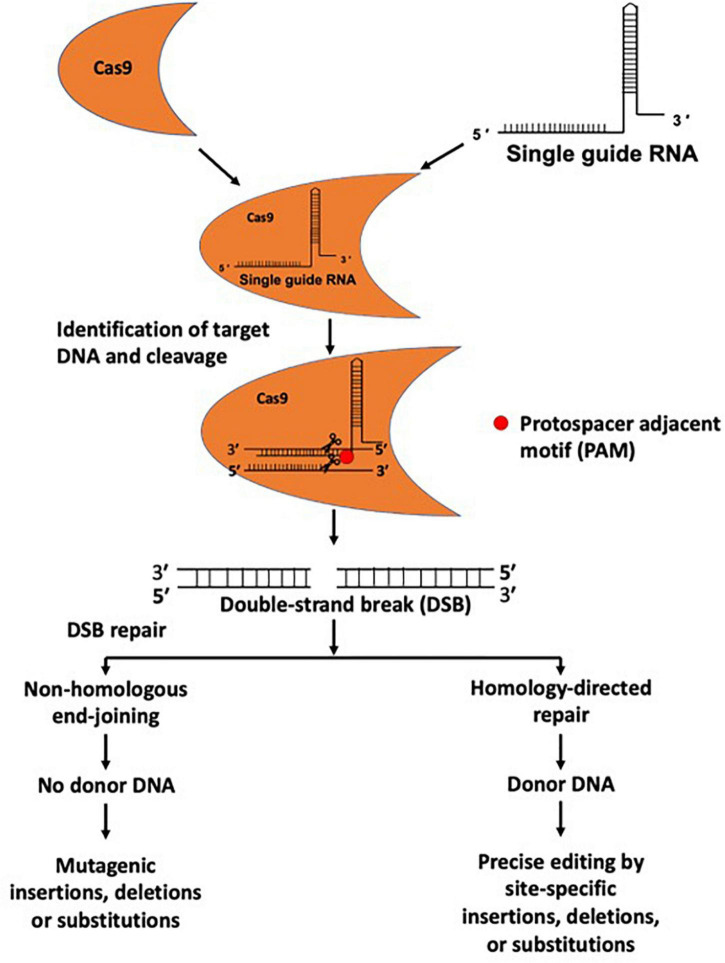
Genome editing using CRISPR/Cas9 system. The CRISPR/Cas9 system is composed of a gRNA and Cas9 protein. gRNAs provide target specificity by sequence complementarity. gRNA and Cas9 proteins form a complex and cleave target DNA at a specific site and produce a double-strand DNA break (DSB). DSBs are repaired through non-homologous end joining (NHEJ) and homology-directed repair (HDR) mechanisms, and during the repair process insertions, deletions, nucleotide substitutions, or gene insertion may occur.

A gRNA has two components, including CRISPR RNA (crRNA) and trans-activating CRISPR RNA (tracrRNA). crRNA contains a 20-nucleotide (nt) RNA sequence complementary to the target DNA sequence, and tracrRNA serves as a Cas nuclease-binding scaffold (Marx, 2020). A single guide RNA (sgRNA) can be generated by combining crRNA and tracrRNA for targeting the gene sequence in the gene-editing tool (Allen et al., 2020). The targeting specificity of Cas9 enables avoidance of off-target effects, which is facilitated by the gRNA sequence (20 nt) (Zhang et al., 2015). Cas9 also requires a specific protospacer adjacent motif (PAM) localized on the non-target strand of DNA directly downstream of the target sequence (Figure 1). However, the possibility of off-target effects due to Cas9 binding to unintended genomic sites for cleavage cannot be entirely ruled out (Zhang et al., 2015; Naeem et al., 2020).

The Cas9 enzyme cuts both complementary and non-complementary strands, causing a double-strand DNA break (DSB), in turn initiating the DNA repair mechanism via error-prone non-homologous end-joining (NHEJ) or precise homology-directed repair (HDR) (Doudna and Charpentier, 2014; Figure 1). NHEJ-induced repair can cause undesirable errors at the target DNA locus, leading to mutagenic insertions and deletions (indels). In contrast, in the presence of a homologous donor template, HDR can cause site-specific insertions, deletions, nucleotide substitutions, or genomic sequence rearrangements, highlighting its potential application for accurate genetic editing (Figure 1). CRISPR/Cas9-based genome editing in eukaryotic cells was first reported in 2013 (Cong et al., 2013). The CRISPR/Cas9 system has become a widely applicable genome editing tool, and its use is not limited to cellular organisms, as it is frequently used in acellular organisms, such as viruses (White et al., 2015; Xiao et al., 2019; Teng et al., 2021).

Hepatitis B virus (HBV) infection is a major global public health concern. HBV can cause a spectrum of illnesses in humans, including acute hepatitis, chronic hepatitis, liver cirrhosis, and hepatocellular carcinoma (HCC) (Liang, 2009). HBV is a partially double-stranded relaxed circular DNA (rcDNA) virus with a genome of 3.2 kb, with four overlapping open reading frames encoding seven viral proteins, including polymerase/reverse transcriptase, core, precore, three related envelope proteins [HBV surface proteins (HBs); large, middle, and small], and a regulatory X protein (Hu and Seeger, 2015; Seeger and Mason, 2015). Despite the availability of an effective preventive HBV vaccine, chronic HBV infection remains a major global health problem affecting millions of people globally (Mastrodomenico et al., 2021; World Health Organization, 2021). A recent estimate indicated that 296 million people were chronically infected with HBV in 2019 (World Health Organization, 2021). Current therapies consisting of pegylated interferon alpha and nucleos(t)ide analogs are only effective at suppressing HBV replication and reducing the risk of cirrhosis, liver failure, and HCC development; however, they cannot cure HBV infection owing to the persistent nature of covalently closed circular DNA (cccDNA) and/or integrated HBV DNA in hepatocytes (Suk-Fong Lok, 2019; Ezzikouri et al., 2020). cccDNA, a replicative template for HBV, must be fully eradicated to completely cure HBV (Nassal, 2015). Therefore, novel alternative therapeutic strategies need to be developed to eradicate cccDNA with minimal side effects, and the CRISPR/Cas system appears to be a promising tool for achieving the goal.

**FIGURE 2:**
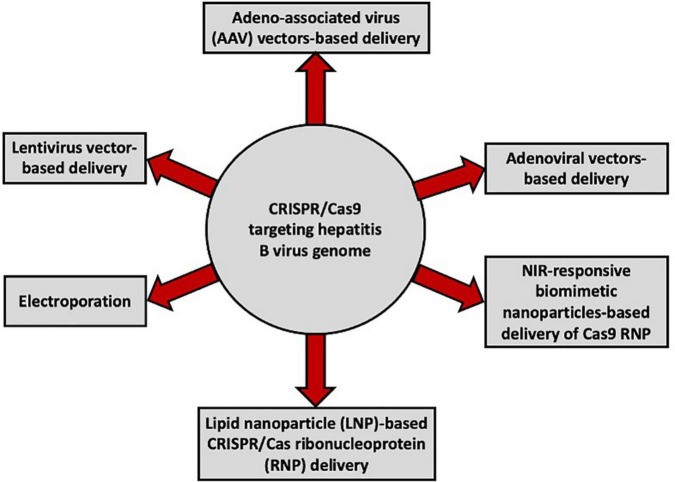
Vectors used for the delivery of CRISPR/Cas9 targeting the HBV genome in different *in vitro* and *in vivo* studies.

With the advent of the CRISPR/Cas technology, several studies have reported varying degrees of inhibition of HBV replication and/or cccDNA formation using the CRISPR/Cas9 system with different delivery tools (Figure 2; Lin et al., 2014; Seeger and Sohn, 2014; Dong et al., 2015; Lin et al., 2015; Liu et al., 2015, 2018; Ramanan et al., 2015; Wang et al., 2015; Zhen et al., 2015; Li H. et al., 2016; Seeger and Sohn, 2016; Zhu et al., 2016; Li et al., 2017, 2018; Kostyusheva et al., 2019; Kayesh et al., 2020; Suzuki et al., 2021; Yan et al., 2021; Martinez et al., 2022; Wang D. et al., 2022). Notably, it is difficult to efficiently quantify cccDNA (Wang Z. et al., 2022), and accurate quantification of intrahepatic cccDNA is important for assessing the efficiency of anti-HBV therapy. Furthermore, in addition to xenografted mice, murine models are extremely inefficient in cccDNA production (Ortega-Prieto et al., 2019; Lai et al., 2021).

In the case of *in vivo* clinical applications, genome editing using the CRISPR/Cas system requires an efficient and reliable delivery tool for transporting CRISPR/Cas into target cells or organs. Different delivery tools, such as physical, chemical, or biological methods, can be used to deliver CRISPR/Cas9 to the host target site. The components of the CRISPR/Cas9 genome editing system are delivered in one of three forms: DNA, RNA, or ribonucleoprotein (RNP) (Luther et al., 2018; Eoh and Gu, 2019; Yip, 2020). However, a practical, safe, and effective method of delivering genome-editing components for *in vivo* genome editing and gene therapy against HBV infection has not yet been developed. Although different delivery tools, such as viral and non-viral tools, are under development (Xu et al., 2021), extensive investigations on potential *in vivo* animal models are required to devise suitable delivery tools to address the issues impeding the clinical application of the CRISPR/Cas system in the treatment of chronic hepatitis B (CHB). Therefore, CRISPR/Cas9 delivery systems with low immunogenicity and high efficiency are required. Moreover, the low efficiency of *in vivo* delivery must be improved before it can be effectively used in therapy (Behr et al., 2021; Taha et al., 2022). Delivery plays an important role in the CRISPR/Cas-mediated inhibition of HBV replication (Suzuki et al., 2021). Against such a background, in the present review, we provide an overview of CRISPR/Cas9-mediated inhibition of HBV, with major focus on delivery systems, particularly adeno-associated virus (AAV) vector-based delivery of CRISPR/Cas9 targeting HBV. In addition, we discuss other tools, including non-viral delivery tools.

## Viral Vectors for Clustered Regularly Interspaced Short Palindromic Repeat/Associated Protein 9 Delivery

Viral vectors are considered some the most promising delivery tools for gene therapy considering their relatively small genome size, genome organization, and evolutionary plasticity (Lukashev and Zamyatnin, 2016). They have shown great promise for application in the delivery of CRISPR/Cas9 for gene editing (Taha et al., 2022). Different viral vectors, including AAV, full-sized adenovirus, and lentivirus vectors, have been investigated for CRISPR/Cas9 delivery (Lino et al., 2018), with varying degrees of success. However, viral vector applications are limited by a number of issues, such as their oncogenic effects, toxicity, immunogenicity, and insertional mutagenesis, which should be addressed in future investigations.

## Adeno-Associated Virus Vector-Based Delivery of Clustered Regularly Interspaced Short Palindromic Repeat/Associated Protein 9 to Target Hepatitis B Virus

**FIGURE 3:**
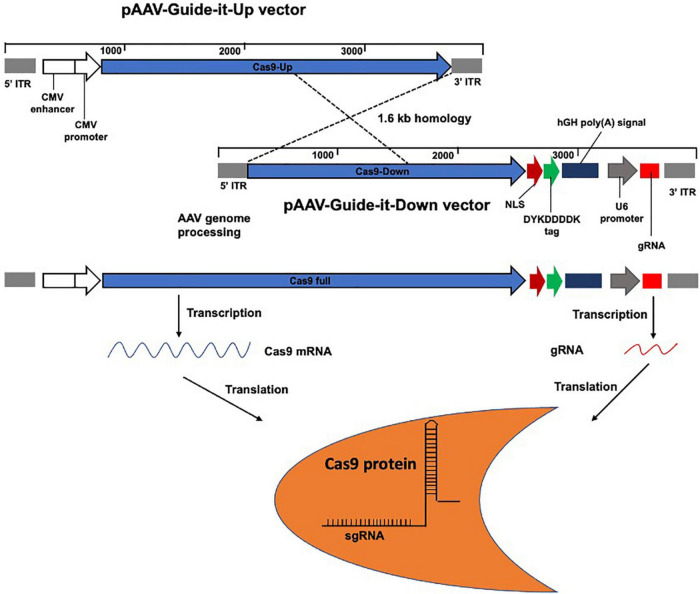
Schematic representation of the larger transgene *Streptococcus pyogenes* Cas9 (SpCas9) incorporation by splitting into AAV vectors. Two vectors pAAV-Guide-it-Up and Guide-it-Down were used to express full-length SpCas9 and gRNA. gRNA sequence is located downstream of the U6 promoter of the Guide-it-Down vector for binding to the target DNA sequence.

AAVs are the most common viral vectors currently being investigated for application in *in vivo* gene therapy, owing to their properties, such as high titer, low immunogenicity, transduction of a broad range of target tissues, and low genomic integration rate (Colella et al., 2018). AAV is one of the most commonly used viral vector systems to date and has attracted considerable attention for use in *in vivo* CRISPR/Cas delivery. AAVs are small viruses that require the presence of a helper virus, including adenoviruses, herpes simplex virus, vaccinia virus, or human papillomavirus, to achieve a productive replication cycle (Geoffroy and Salvetti, 2005). AAV belongs to the family *Parvoviridae* and genus *Dependovirus* (Daya and Berns, 2008). They are single-stranded DNA viruses with a genome of approximately 4.7 kilobases (Wu et al., 2010). The AAV genome encodes several proteins, including four non-structural Rep proteins (Rep78, Rep68, Rep52, and Rep40) required for viral replication, three capsid proteins (VP1–VP3), and an assembly-activating protein (Sonntag et al., 2010). To date, 12 human serotypes of AAV, designated AAV-1 to AAV-12, and over 100 serotypes in non-human primates, have been identified. Nearly all AAV serotypes exhibit natural hepatic tropism and efficiently accumulate in the liver following intravenous administration (Palaschak et al., 2019). AAV vectors have been successfully used to treat patients with bleeding disorders and blindness (Nathwani et al., 2011; Tuddenham, 2012; Vandenberghe and Auricchio, 2012; George et al., 2017; Li and Samulski, 2020). Although AAV vectors are restricted by their limited packaging capacity (Grieger and Samulski, 2005), by using dual AAV vectors or triple AAV vectors, the transfer capacity of AAV can be expanded from 4.7 kb to approximately 9 or 14 kb, respectively (Yang et al., 2016; Bak and Porteus, 2017; Maddalena et al., 2018). One strategy of overcoming the size limit of AAV vectors is splitting large transgenes into two or three parts to generate dual or triple AAV vectors (Akil, 2020). Transduction of target cells with these two or three AAVs, through homologous recombination or other mechanisms, results in the transcription of a full-length Cas9 mRNA (Bak and Porteus, 2017; Akil, 2020). In our previous study, full-length *Streptococcus pyogenes* Cas9 (SpCas9) was obtained by splitting the SpCas9 transgene using dual AAVs (Figure 3) modified from a previous study (Kayesh et al., 2020). Moreover, a smaller Cas9, *Staphylococcus aureus* Cas9 (SaCas9), has been discovered, containing 1,082 amino acid residues, with 286 residues less than that of SpCas9, and has been used for delivery by AAV vectors. However, delivery with dual AAVs requires a high viral dose, which raises potential safety concerns and can reduce the editing potential (Hayashi et al., 2020; Fang et al., 2021). Although most AAV vectors exist in an extrachromosomal state, a fraction of AAV vectors can integrate into pre-existing DSBs (Miller et al., 2003, 2004). Notably, a recent study has reported high levels of AAV integration (up to 47%) in Cas9-induced DSBs (Hanlon et al., 2019). However, genome integration is greatly reduced in recombinant AAVs (rAAVs), which are devoid of the rep gene, and ITRs are the only viral origin sequences used to guide genome replication and packaging during vector production (Wang et al., 2019). As prolonged expression of Cas9 may increase the possibility of off-target effects, which can cause safety concerns, delivery in DNA form is advantageous if sustained Cas9 expression is required (Ishida et al., 2015; Yip, 2020).

Cas9 is an endonuclease that contains two nuclease domains, RuvC and HNH. The RuvC domain cleaves non-complementary DNA strands and the HNH domain cleaves complementary DNA strands (Liu et al., 2017). The SpCas9 is the most widely used endonuclease and has been demonstrated to effectively inactivate HBV sequences (Hille et al., 2018; Moyo et al., 2018). However, different Cas9 orthologs, such as SaCas9, *Streptococcus thermophilus* Cas9 (StCas9), *Neisseria meningitidis* Cas9 (NmCas9), and SpCas9 variants, have been optimized (Kleinstiver et al., 2015). A recent study reported preexisting adaptive immune responses in humans against SaCas9 and SpCas9 proteins (Simhadri et al., 2018; Charlesworth et al., 2019; Wagner et al., 2019), suggesting previous exposure of these bacterial Cas9 proteins from microbes in humans. Pre-existing immunity against Cas9 may negatively impact its clinical use by affecting its efficacy and posing significant safety issues (Mehta and Merkel, 2020). Therefore, it is essential to fully characterize the impact of pre-existing immunity against Cas9 for its successful use in *in vivo* genome editing. SaCas9 and sgRNA-8 delivered using single-stranded adeno-associated viral vectors (ssAAVs) resulted in HBV DNA and cccDNA suppression in HepG2.2.15 and HepG2-hNTCP cells (Scott et al., 2017). gRNA/SaCas9 delivered by AAV inhibited HBV antigen, pgRNA, and cccDNA in different cell lines, including Huh7, HepG2.2.15, and HepAD38 cells (Liu et al., 2018). As expected, the dual expression of gRNAs/SaCas9 was more efficient for HBV genome cleavage (Liu et al., 2018). Although not significant, the levels of HBsAg, HBV DNA, and pgRNA were decreased in mice with persistent HBV replication and a higher titer of AAV injection when compared with that in controls (Liu et al., 2018). In addition, a recent study showed inhibition of HBV by CRISPR/SaCas9 delivered using hepatotropic AAV8 in C57BL/6 mice (Yan et al., 2021). In another study, C57BL/6 mice were administered 2 × 10^11^ AAV8 vector genomes (vg) via tail vein injection to deliver SaCas9 in a volume of 200 μL (Yan et al., 2021).

CRISPR generates knockouts at the DNA level, whereas RNA interference (RNAi) silences genes by generating knockdowns at the mRNA level. RNAi has been shown to exert antiviral effects against HBV (Mccaffrey et al., 2003). A single administration of a double-stranded AAV8-pseudotyped vector (dsAAV2/8) carrying HBV-specific shRNA reportedly effectively suppressed HBV protein, mRNA, and replicative DNA in the liver of HBV transgenic mice, and the effect was sustained for at least 120 days after vector administration (Chen et al., 2007). However, the suitability of the dsAAV2/8 vector for CRISPR/Cas9-based therapies and the treatment of chronic HBV infection remains to be investigated. Notably, AAV vectors obtained using the pseudotyping method are often referred to as AAV2/n, where the first number refers to the ITRs and the second to the capsid. Capsids are responsible for different transduction abilities (i.e., cell tropism and kinetics of transgene expression), and users can choose accordingly (Asokan et al., 2012; Balakrishnan and Jayandharan, 2014). Varying hepatocyte transduction efficiencies have been reported among AAV serotypes, and an engineered AAV3 capsid has been reported to exhibit much greater efficiency for human hepatocytes than AAV5, AAV8, and AAV9 (Vercauteren et al., 2016). Another recent study reported a mutant AAV vector candidate, AAV3B, with greater hepatocyte tropism and reduced seroreactivity (Biswas et al., 2020). Development of mutant AAV variants with human hepatocyte tropism and neutralizing antibody escape capacity is paramount, and further investigation is required to obtain a suitable vector for CRISPR/Cas9-mediated inhibition of HBV infection (Pei et al., 2020).

In our previous study, we investigated the potential of AAV2-mediated delivery of guide (g) RNAs/Cas9 to inhibit HBV replication both *in vitro* and *in vivo*. For *in vivo* experiments, 1 × 10^12^ vg copies of AAV2-WJ11/Cas9 were injected through the tail vein into persistent HBV genotype C-infected humanized chimeric mice. We observed that AAV2-WJ11/Cas9 significantly inhibited HBV DNA replication and reduced cccDNA levels both *in vitro* and *in vivo* (Kayesh et al., 2020); however, the effect required high multiplicity of genome (MOG) copies of the AAV2 vector, suggesting low transduction efficiency of the AAV2 vector. Using an immunocompetent tree shrew model of HBV infection (Kayesh et al., 2021), we observed a much lower transduction efficiency of the AAV2 vector in liver tissues (unpublished data) than in humanized chimeric mice (Kayesh et al., 2020). Stone et al. demonstrated the antiviral efficacy of SaCas9 in humanized FRG mice chronically infected with HBV genotype C, which resulted in decreased total liver HBV DNA and cccDNA levels (Stone et al., 2021). Mice were administered with 5 × 10^11^ AAV vector genome copies generated with capsid LK03 via tail vein injection to deliver SaCas9 in a volume of 100 μL (Stone et al., 2021). Although AAV is one of the most commonly used vector systems, a very high dose is required for its transduction (Kayesh et al., 2020). Despite years of research, AAV production is still expensive and remains one of the main barriers for AAV-based gene therapy (Naso et al., 2017; Wang et al., 2019), making it impractical to implement programs to treat HBV-infected individuals with AAVs globally. Moreover, problems such as liver toxicity at high doses, immunogenicity, off-target effects, and genomic integration remain to be resolved.

## Adenoviral Vectors as a Delivery Tool for Gene Therapy

Adenoviruses belong to the *Adenoviridae* family, and comprise a wide group of human and animal viruses that share functional, genetic, and structural characteristics (Smith et al., 2010). Adenoviruses have a linear double-stranded DNA genome that ranges from 26 to 46 kb in length (San Martin, 2012). The adenovirus genome encodes more than 40 different proteins, 12 of which are mature viral particles (Lehmberg et al., 1999). The episomal nature, large cloning capacity, high titers, and transducing ability of dividing and non-dividing cells make adenoviral vectors (AdVs) interesting candidates for exploitation in RNA-guided nuclease (RGN) delivery (Goncalves and De Vries, 2006). High-capacity adenoviral vectors (HCAdVs) lacking all the coding genes are considered powerful tools for the delivery of large amounts of DNA cargo into cells (Ehrke-Schulz et al., 2017). HCAdVs possess high packaging capacity (up to 35 kb), low immunogenicity, and low toxicity (Xu et al., 2019). Schiwon et al. (2018) demonstrated that co-delivery of multiple gRNA expression cassettes along with the Cas9 expression cassette through one HCAdV resulted in a significant reduction in HBV Ag and HBV cccDNA production.

## Lentivirus Vector-Based Delivery of Clustered Regularly Interspaced Short Palindromic Repeat/Associated Protein 9 for Hepatitis B Virus Inhibition

Lentiviral vector (LV) is a single-stranded RNA virus with a packaging capacity of approximately 8 kb (Vogt and Simon, 1999). Although LVs can mediate potent transduction and stable expression in dividing and non-dividing cells both *in vitro* and *in vivo*, their use in clinical research is limited by several issues related to safety, ethics, and public health concerns, and significantly lower transduction efficiency in non-dividing cells that are quiescent in the G0 state (Connolly, 2002; Kuhn et al., 2002). To address such challenges, non-integrating lentiviral vectors (NILVs) are under development (Uchida et al., 2021).

LV-mediated delivery of CRISPR/Cas9 has been shown to efficiently cleave viral DNA and suppress HBV in HepG2.2.15 and HepG2-hNTCP cells (Ramanan et al., 2015). Dual expression of gRNA/CRISPR/Cas9 results in greater reductions in HBsAg and HBV RNA when compared with the expression of single guide RNAs (Ramanan et al., 2015). An anti-HBV effect in a mouse model was observed upon introduction of HBV and Cas9/sgRNA plasmids into the liver of immunodeficient mice (NRG) by hydrodynamic injection (HDI) (Ramanan et al., 2015), which suggests the efficacy of CRISPR/Cas9 system-based targeting of the HBV genome. Another study identified conserved HBV sequences in the S and X regions of the HBV genome, which were targeted for precise and effective cleavage by Cas9 nickase (Karimova et al., 2015). Base editing has advanced CRISPR/Cas-based technologies, and can be used to directly initiate point mutations in cellular DNA without a DSB (Kantor et al., 2020). The CRISPR/Cas9-mediated non-cutting editing strategy in the base-editing system has been shown to have the potential to cure CHB by permanent inactivation of integrated HBV DNA and cccDNA without off-target effects (Yang et al., 2020). Another recent study also reported efficient silencing of HBV following targeting of the HBV S gene using CRISPR-mediated base editing (Zhou et al., 2022).

## Non-Viral Vectors for Clustered Regularly Interspaced Short Palindromic Repeat/Associated Protein 9 Delivery

Although viral vector-mediated gene delivery results in higher transduction efficiency and long-term gene expression, viral vectors suffer from a number of limitations, including oncogenic effects, toxicity, immunogenicity, poor target cell specificity, inability to transfer large genes, insertional mutagenesis, and high costs (Wang et al., 2013). Non-viral vectors are based on the collective delivery of naked RNA or DNA using chemical or physical methods, resulting in efficient delivery of nucleic acids into the target cells (Wang et al., 2013; Jin et al., 2014). Non-viral vectors, particularly cationic lipid-based biomaterial approaches, show high potential because of their relative safety, ease of preparation, cell/tissue targeting, and low immunogenicity. However, the clinical application of non-viral methods is limited by their low transfection efficiency and poor transgene expression (Wang et al., 2013). In recent years, non-viral vectors have been found to be effective for CRISPR/Cas9 delivery to cells and tissues *in vivo* and *in vitro*. Various promising non-viral tools for CRISPR/Cas9 gene therapy have been developed, including liposomes (Liu et al., 2019), nanocarriers (Miller et al., 2017; Finn et al., 2018), and cell-penetrating peptides (Suresh et al., 2017). Nanoparticles targeting hepatocytes have been developed using endogenous and exogenous targeting ligand-based mechanisms using glycans, proteins, or modifications of the nanoparticle surface (Barrett et al., 2014; Detampel et al., 2014; Li M. et al., 2016). In the case of CRISPR/Cas9 gene therapy against HBV using a non-viral vector as a delivery tool, strong hepatotropism of the vector is essential for targeting the liver. In addition, lipid nanoparticles (LNPs) have been found to be efficient carriers of short-interfering RNAs (siRNAs) to hepatocytes *in vivo* (Akinc et al., 2010), which suggests the hepatotropism of LNPs.

A previous study showed that CRISPR/Cas9-delivered by lipid-like nanoparticles (LLNs) (Li et al., 2015) could suppress HBV DNA in a mouse model (Jiang et al., 2017). In a previous study, we also developed LNP as a non-viral delivery tool to deliver the CRISPR/Cas9 system and guide RNA, and investigated its usefulness in CRISPR/Cas9-mediated inhibition of HBV in HBV-replicating HepG2.2.15 cells (Suzuki et al., 2021). Furthermore, LNP-based CRISPR/Cas RNP delivery has been found to significantly enhance the inhibition of *in vitro* HBV replication (Suzuki et al., 2021) when compared with viral vector (AAV2) delivery (Kayesh et al., 2020), highlighting the relevance of the delivery tool in enhancing antiviral effects. Another recent study showed that the RNP delivery of CRISPR/Cas9 in HBV-infected HepG2-NTCP cells induced DSBs in cccDNA, which affected HBV replication. Moreover, Cas9-induced effects were sustained even after RNP degradation/loss of detection, suggesting stable changes due to transcriptional interference (Martinez et al., 2022). However, the efficacy of LNP-based CRISPR/Cas RNP delivery targeting the HBV genome remains to be investigated using a bona fide *in vivo* HBV infection model.

A previous study reported the synthesis and development of zwitterionic amino lipids (ZALs) that can co/deliver long RNAs, including Cas9 mRNA and sgRNAs (Miller et al., 2017). However, their use in the delivery of CRISPR/Cas9 to target the HBV genome remains to be investigated. A near-infrared (NIR) light-responsive nanocarrier, CRISPR/Cas9, was developed to target cancer therapeutics using upconversion nanoparticles (UCNPs) (Pan et al., 2019). NIR-responsive biomimetic nanoparticle (UCNPs-Cas9@CM)-based delivery of Cas9 RNP was found to inhibit HBsAg, HBeAg, HBV pgRNA, HBV DNA, and cccDNA both *in vitro* (HBV-infected cells) and *in vivo* (HBV-Tg mice) (Wang D. et al., 2022). A recent study reviewed the suitability and efficiency of nanoparticle-based delivery of CRISPR/Cas9 for genome editing (Duan et al., 2021), and the study could facilitate the selection of nanoparticle-based delivery of CRISPR/Cas9 to target HBV infections. Although CRISPR/Cas9 has been employed in clinical trials targeting host genes or viruses, to date, no study has been conducted to target the HBV genome using CRISPR/Cas9 (ClinicalTrials.gov; accessed on May 20, 2022), suggesting that further expanded preclinical investigations are still required before engaging in clinical trials.

## Physical Methods for Clustered Regularly Interspaced Short Palindromic Repeat/Associated Protein 9 Delivery

### Electroporation

Electroporation is widely used to deliver nucleic acids and proteins to mammalian cells (Mali et al., 2013; Tebas et al., 2014). Although electroporation can be used to deliver all types of CRISPR/Cas9 systems, including plasmid-based CRISPR/Cas9, Cas9 mRNA and sgRNA, and Cas9/sgRNA RNP, its use is limited because of the low plasmid DNA integration (approximately 0.01% of the target cells) and induction of significant cell death (Liu et al., 2017). Recently, Zhen et al. (2021) reported that combination therapy with anti-HBV and anti-PD1 gRNA/cas9 delivered using electroporation produces a synergistic antiviral effect against HBV infection.

Based on the available data and findings, the main advantages and disadvantages of various tools that have been investigated to date for the delivery of CRISPR/Cas9 targeting the HBV genome are summarized in Table 1.

**TABLE 1:** Advantages and disadvantages of the various delivery tools used for CRISPR/Cas9 delivery.

Delivery tools	Advantages	Disadvantages
Adeno-associated virus vectors	• Widely studied • Safe • Broad tissue tropism, and some AAV serotypes, including AAV8, AAV8-pseudotyped vector (dsAAV2/8), and AAV3B exhibit high hepatocyte tropism	• Difficulty to produce • Limited packaging capacity • Low transduction efficiency • Serotype-dependent preexisting immunity • Repeated injections may be required • Liver toxicity at high dose (>10^14^ vg/kg)
Lentivirus vectors	• High transduction efficiency • Long-term gene expression	• Can cause insertional mutagenesis • Do not efficiently transduce quiescent (G0) cells in the adult liver
Adenovirus vectors	• High infection efficiency • Large cloning capacity • Ability to transduce both dividing and non-dividing cells • Can be produced at high titer	• Transient expression • Toxicity at high dose • Serotype-dependent preexisting immunity • Repeated injections may be required • Induction of both innate and adaptive immune response
Lipid nanoparticle (LNP)-based CRISPR/Cas ribonucleoprotein (RNP)	• Easy scalable production • High delivery efficiency • Low toxicity • Transient expression resulting in lower off-target risk	• Repeated injections may be required • Poor efficiency in penetrating into the nucleus
NIR-responsive biomimetic nanoparticles	• Minimal off-target effects • Good biocompatibility	• Multiple interactions are required • Increased concentrations can cause cytotoxicity
Electroporation	• Suitable for all cell types • High transfection efficiency • Suitable for all CRISPR/Cas9 strategies	• Can cause significant cell death • Non-specific transfection

## Conclusion

A suitable delivery tool is crucial for the achievement of the desired CRISPR/Cas9 effects against HBV infection. Therapeutic translation of the CRISPR/Cas system remains a challenge because of the lack of a suitable delivery tool (Tong et al., 2019; Wilbie et al., 2019). Researchers are actively pursuing the development of efficient CRISPR/Cas delivery systems, which may address the issue in the near future. Although AAVs are the most widely investigated delivery tools for CRISPR/Cas9 targeting of the HBV genome, no clinical trials have been conducted to date with AAVs. Non-viral nanoparticle-based delivery tools, which may supersede the use of viral vectors in the near future, can be considered for extensive future investigations and could open new avenues for nanoparticle-based effective delivery of CRISPR/Cas9 against HBV infection.

## Author Contributions

MEHK, MK, and KT-K: conceptualization. MEHK and KT-K: writing—original draft preparation. MEHK, MH, MK, and KT-K: writing—review and editing. All authors have read and agreed to the published version of the manuscript.

## Conflict of Interest

The authors declare that the research was conducted in the absence of any commercial or financial relationships that could be construed as a potential conflict of interest.

## Publisher’s Note

All claims expressed in this article are solely those of the authors and do not necessarily represent those of their affiliated organizations, or those of the publisher, the editors and the reviewers. Any product that may be evaluated in this article, or claim that may be made by its manufacturer, is not guaranteed or endorsed by the publisher.
